# Adipose Stem Cells (ASCs) and Stromal Vascular Fraction (SVF) as a Potential Therapy in Combating (COVID-19)-Disease

**DOI:** 10.14336/AD.2020.0422

**Published:** 2020-04-22

**Authors:** Pietro Gentile, Aris Sterodimas

**Affiliations:** ^1^Department of Surgical Science, University of Rome Tor Vergata, Rome, Italy; ^2^Department of Plastic and Reconstructive Surgery, Metropolitan General Hospital, Athens, Greece

**Keywords:** coronavirus, mesenchymal stem cells, Covid-19, adipose derived stem cells, Adipocyte secreted exosomal microRNA

## Abstract

A recent and interesting study reported improved respiratory activity after intravenous administration of mesenchymal stem cells (MSCs) into patients affected by coronavirus disease 2019 (COVID-19). These outcomes displayed that intravenous infiltration of MSCs is a safe and efficacy treatment for COVID-19 pneumonia, a severe acute respiratory illness caused by the coronavirus 2 (SARS-CoV-2). Only 7 patients were treated, but with extraordinary results, opening a new strategy in COVID-19 therapy. Currently, no specific therapies against SARS-CoV-2 are available. The MSCs therapy outcomes reported, are striking, as these cells inhibit the over-activation of the immune system, promoting endogenous repair, by improving the lung microenvironment after the SARS-CoV-2 infection. The MSCs could represent an effective, autologous and safe therapy, and therefore, sharing these published results, here is reported the potential use possibilities in COVID-19 of the most common MSCs represented by Adipose Stem Cells (ASCs).

## Introduction

Robert Chunhua Zhao's group [1] reported in a recent study, published in March 2020, an interesting improvement in pulmonary functional activity, into 7 patients affected by Coronavirus Disease 2019 (COVID-19) after a intravenous administration of clinical-grade mesenchymal stem cells (MSCs). The COVID-19, as already known, is a severe acute respiratory illness caused by a new coronavirus named coronavirus 2 (CoV-2) [2-3]. Cov-2 is first appeared in Wuhan, China, but has now disseminated to multiple countries in the world, including Europe, the United Kingdom, and the United States [2-3]. In Europe, the situation is most serious in Italy (www.salute.gov.it) and Spain, while currently the United States seems to be the most affected country in the world.

It is not clear the origin of the virus. Several hypotheses have been reported, without any scientific proof.

Zhou P. *et al.* [4] in an article published in Nature, Mar 12, 2020, reported that the sequences of 2019-nCoV (SARS-Cov-2) were almost identical and share 79.6% sequence identity to SARS-CoV. In detail, they confirmed that 2019-nCoV uses the same cell entry receptor-angiotensin converting enzyme II (ACE2) as SARS-CoV [4]. They clearly affirm, *"We do not yet know the transmission routine of this virus among hosts... Owing to a shortage of specific treatments and considering the relatedness of 2019-nCoV to SARS-CoV, some drugs and pre-clinical vaccines against, SARS-CoV could probably be used to treat this virus"* [4].

At the current time, there is no scientific evidence of the first transmission kind, as previously reported, while other notices are mediatic only.

Today, April 22, 2020, as reported by www.worldometers.info/coronavirus/ we have in the World 2,572,805 Coronavirus Cases, 178,551 deaths and 701,552 recovered.

For this reason, it appears to be necessary very quickly test new therapies.

In the ongoing pandemic, the only means available to reduce the infection are represented by travel restrictions, containment measures, and mitigation strategies or as currently in Italy and Spain, the total lockdown.

Starting with the preliminary and precious experience published by Zhao's group [1] we aim to suggest an immediate, easy and safe therapy in Covid-19 patients via the most common and important MSCs represented by ASCs so as to help the research.

### Intravenous Infusion of Mesenchymal Stem Cells (MSCs) in Covid-19 patients

In the preliminary study of Robert Chunhua Zhao's group [1], 7 patients affected by SARS-CoV-2, with COVID-19 pneumonia displayed a sensible improvement pulmonary function after several intravenous infusion of clinical-grade MSCs [1]. The results were compared with a little control group composed of 3 patients treated with a placebo.

The authors described the preparation of infusion suspension, suspending a cellular product of MSCs in 100 mL of saline, and reporting the total amount of infused cells was 1 × 10^6^ cells per kg. The infusion was performed for about 40 minutes with a speed of ~40 drops per minute when symptoms still were getting worse [1], then the patients have been observed closely for 14 days. Surprisingly, the study reported that all pulmonary and other symptoms subsided 3 days average later receiving intravenous MSCs infusion without side effects. Extraordinarily, the post-treatment CT imaging showed that pneumonia was significantly decreased, and the major part of treated patients had shown negative results for the SARS-CoV-2 nucleic acid test 1.5 weeks average later the treatment [1].

Starting with this fundamental work, it is necessary to specify that, as reported in the study [1], the MSCs used, were a certified cellular product.

The rationale of the present work is to suggest the possibility to use autologous or allogeneic ASCs intravenously or directly through a ventilation mask (aerosol). Autologous ASCs can be collected and infused quickly while for allogeneic ASCs it is necessary to have a Good Manufacturing Practices (GMP) lab approval.

### The potential use of MSCs and ASCs, and their bio-molecular implications in COVID-19

MSCs have been used extensively in cellular therapies, including both pre-clinical and clinical studies [5-8], suggesting their safety and efficacy. The sources of MSCs are principally two: adipose tissue, and bone marrow [9]. Recent studies introduced a new, interesting, and alternative sources of MSCs represented by Dental Pulp Stem Cells (DPSCs) [10], Periodontal Ligament stem cells (PDLSCs) [11], and Oral Stem Cells [12]. The sub-cutaneous adipose tissue offers a significant edge over other sources because it is easily accessible posing the least amount of discomfort to the patient and being easy to use with local anesthesia. Additionally, it is extremely easy to isolate the stem cells from the adipose tissue via minimal manipulation or enzymatic digestion of fat [13, 14].

Minimal manipulation is based on fat mechanical centrifugation and/or filtration, while enzymatic digestion on collagenases uses, as previously described [9, 15-21]. A difference in terms of stem cell amount was reported between procedures [9, 15-21].

A higher quantity of stem cells was observed in adipose tissue compared to bone marrow [22]. MSCs are multi-potent cells that renew on their own, having the capacity to split into cells of mesenchymal origin *in vitro* as chondrocytes, adipocytes, and osteoblasts. Human ASCs express the classical mesenchymal markers such as CD44, CD73, CD90, CD105 and CD166 [9], and are located in Stromal Vascular Fraction (SVF) portion of sub-cutaneous fat [23]. For these reasons, it is possible to identify the ASCs as the most important representative of MSCs.

MSCs and ASCs with related SVF portion have been used in the immune-mediated inflammatory diseases (graft-versus-host disease and systemic lupus erythematosus) [24, 25], in lower extremity ulcers [26], calvarial defects [27], craniofacial microsomia [28], breast reconstruction [16-19, 29, 30], outcomes of burns and scars [20] with safe and interesting results.

The improved pulmonary and other organs function after MSC infusions in the study of Zhao's group [1], can be attributed both to immune-modulatory that also to the anti-inflammatory MSCs effects, as these cells release many paracrine factors, which interact with immune cells resulting in immunomodulation, and with inflammation decrease [5, 6, 31]. These effects were confirmed by the increased peripheral lymphocytes amount, the decline in the C-reactive protein, and waning of over-activated cytokine-secreting immune cells (CXCR3+CD4+ T cells, CXCR3+CD8+ T cells, and CXCR3+ NK cells) into the blood of treated patients, by mean 4.5 days later the intravenous infusion [1]. Moreover, 10 x RNA-sequencing analysis displayed that infused MSCs were negative for ACE2 and TMPRSS2, which confirmed that these cells were free from COVID-19 infection. The possible implications of MSCs as anti-viral therapy have been cited by also the Kyoto Encyclopedia of Genes and Genomes (KEGG) [1].

Now, the adipose-derived MSCs and related SVF have been routinely used for many years in autologous regenerative therapies, displaying effective and safe results, as previously cited. They could have also a potential allogeneic use via a specific Human Tissue Fat Bio-Bank that lacks at this moment or via GMP laboratory.

Currently, there are 22 clinical trials registered (https://clinicaltrials.gov) to evaluate the MSCs as clinical treatment of patients affected by COVID-19. (https://clinicaltrials.gov/ct2/results?cond=COVID-19&term=Mesenchymal%20Stem%20Cells&cntry=&state=&city=&dist=). Of these clinical trials, in particular, two are on DPSCs, five are on Umbilical Cord Stem Cells (UC-SCs), one is on Mesenchymal Stromal Cells, one on MSCs-Exosomes and two are on ASCs.

It is too early to know if ASCs will be used as part of future treatment options for COVID-19 or similar conditions with significant complications, but there is the potential that the work we are seeing reported on today will become a part of helping patients with COVID-19. In each case, it is necessary to specify that these procedures are possible only if performed and authorized by the GMP lab or EMA in Europe and by FDA in the United States.

### ASCs and SVF potential effects through secretory and anti-inflammatory activities

As briefly introduced, SVF contains a larger cellular population, represented by ASCs and Stromal vascular Fraction Cells (SVFs). SVFs and ASCs secrete pro-angiogenic factors, such as Vascular Endothelial Growth Factor (VEGF), platelet-derived growth factors (PDGF), inducing proliferation of endothelial cells, promoting the vascularization, providing physical Extracellular Matrix (ECM) guidance cues that promote endothelial sprouting [17, 18]. Moreover, SVFs and ASCs have immune-modulating proprieties mediated by transforming growth factor-1 (TGF-1), hepatocyte growth factors (HGF) and interferon-γ (INF-γ) [17, 18]. These activities and the early establishment of new micro-capillary networks, which deliver the proper nutrients and oxygen, might contributed to the improved outcomes observed during MSCs infusion in Covid-19 patients.

The high secretory activity makes also SVFs and ASCs, as MSCs, a potentially suitable vehicle for the delivery of drugs molecules in the cellular microenvironment, with the potential aim to regenerate damaged tissue as for to nanotechnologies, drug-loaded exosomes, and micro-RNAs (MiRs) [18]. Several MiRs are present in fat, actively participating in the adipogenesis regulation, adipokine secretion, inflammation, and inter-cellular communications in the tissues. These results provide important insights into Adipocyte-secreted exosomal microRNAs (A-Se-MiRs) function and they suggest evaluating the potential role of A-Se-MiRs in human organs and tissue regeneration [18]. In particular, the situation produced by the COVID-19 in currently April, 2020 as pandemic, in which there is not actually any therapy, any vaccines, must push reflect about the idea that may be necessary resort to our SVFs and ASCs and related MiRs for the cure of human pathologies or organ damages.

### Rationale of the ASCs and SVFs use

The adipose-derived MSCs, as ASCs and SVFs in which they are contained (1mL of fat tissue offers 100.000 SVFs of which 1% - 3% are ASCs = 1.000/3.000), can be collected by 100mL of fat tissue, obtained by a very simple, fast and safe gently liposuction, performed also in local anesthesia, from the abdomen, flank and thighs regions [15-17, 20].

With 100mL of processed fat via minimal manipulation, it is possible to obtain an average of 1 × 10^7^ SVFs containing ASCs [15-18].

Robert Chunhua Zhao's group [1] reported the use of 1 × 10^6^ MSCs per Kg of weight. For this reason, it is possible to think that they infused 75 × 10^6^ MSCs (75.000.000 MSCs) for each patient, hypothesizing a weight mean of 75Kg (data not published). Using the minimal manipulation of fat tissue could be necessary to collect a 500-1000mL average of fat tissue, to generate 50 × 10^6^ - 100 × 10^6^ 50.000.000-100.000.000 adipose-derived MSCs. Using enzymatic digestion of fat tissue, it is possible to divide two different procedures: manual and automatic extraction.

From adipose tissue, by manual extraction, it was obtained approximately 250,000 ± 34,782 nucleated SVFs cells per each mL of fat tissue; on the other hand, by the automatic extractor, the cell yield was approximately 50,000 ± 6,956 nucleated SVFs cells per ml of fat tissue (*p* < 0.01) [20]. In the first case, 25 × 10^6^ (25.000.000) nucleated SVFs cells can be obtained by 100mL of fat tissue, and 300mL of fat (average 150-400mL) would be the right amount to obtain average 75 × 10^6^ 75,000,000 cells.

Alternatively, using a few each mL of fat tissue for each patient (5-10mL) would be necessary to perform extensive manipulation via GMP lab, to obtain the same quantity of cells in form of the cellular product like as which used by Zhao's group [1]. In Italy for example, we have 17 GMP authorized lab, but there are not studies published in this sense.

All these procedures of fat tissue manipulation, aimed to obtain an SVFs pellet containing ASCs, are regulated by the European rules (1394/2007 EC) and EMA/CAT recommendations (20 June 2014 EMA/CAT/600280/ 2010 Rev 1) [17, 18, 21].

### Conclusions

It is not more possible to accept the idea, that for a viral pandemic, at the current day, it is necessary to stay at home to avoid contagion, like Middle Ages, or it is necessary to be hospitalized, in intensive therapy to continue to breathe.

For this reason, ASCs, SVFs both autologous and allogeneic, A-Se-MiR and each type of MSCs may offer new and alternative approaches for the Covid-19 therapy. ASCs and SVFs could be infused today quickly and safely.
